# Sudden Cardiac Death—A New Insight Into Potentially Fatal Genetic Markers

**DOI:** 10.3389/fmed.2021.647412

**Published:** 2021-03-22

**Authors:** Dragan Primorac, Ljubica Odak, Vitorio Perić, Jasmina Ćatić, Jozica Šikić, Vjekoslav Radeljić, Šime Manola, Robert Nussbaum, Matteo Vatta, Swaroop Aradhya, Tanja Sofrenović, Vid Matišić, Vilim Molnar, Andrea Skelin, Jure Mirat, Johannes Brachmann

**Affiliations:** ^1^St. Catherine Specialty Hospital, Zagreb, Croatia; ^2^Eberly College of Science, The Pennsylvania State University, University Park, State College, Philadelphia, PA, United States; ^3^The Henry C. Lee College of Criminal Justice and Forensic Sciences, University of New Haven, West Haven, CT, United States; ^4^Medical School, University of Split, Split, Croatia; ^5^Faculty of Dental Medicine and Health, Josip Juraj Strossmayer University of Osijek, Osijek, Croatia; ^6^Faculty of Medicine, Josip Juraj Strossmayer University of Osijek, Osijek, Croatia; ^7^Medical School, University of Rijeka, Rijeka, Croatia; ^8^Medical School REGIOMED, Coburg, Germany; ^9^Medical School, University of Mostar, Mostar, Bosnia and Herzegovina; ^10^Children's Hospital Zagreb, Zagreb, Croatia; ^11^Department of Cardiology, Clinical Hospital Dubrava, Zagreb, Croatia; ^12^Department of Cardiology, Clinical Hospital Sveti Duh, Zagreb, Croatia; ^13^Department of Cardiology, Clinical Hospital Center Sestre Milosrdnice, Zagreb, Croatia; ^14^Invitae, San Francisco, CA, United States

**Keywords:** sudden cardiac death, genes, coronary artery disease, cardiomyopathy, professional athletes

## Abstract

Sudden cardiac death (SCD) is an unexpected and dramatic event. It draws special attention especially in young, seemingly healthy athletes. Our scientific paper is based on the death of a young, 23-year-old professional footballer, who died on the football field after a two-year history of cardiac symptoms. In this study we analyzed clinical, ECG and laboratory data, as well as results of genetic testing analysis in family members. To elucidate potential genetic etiology of SCD in this family, our analysis included 294 genes related to various cardiac conditions.

## Introduction

### Definition of Sudden Cardiac Death

Sudden cardiac death (SCD) is an unforeseen, unexpected heart function loss that occurs within 1 h of symptom onset or within 24 h of last being seen alive (1). SCD may occur after a long history of cardiac disease, but it can also be the first manifestation of genetic heart disease in a seemingly healthy person (1, 2). The cause of death is either cessation of heartbeat or irregular heart rhythm, both resulting in decreased tissue perfusion.

### SCD in the Population: Epidemiology and Etiology

SCD presents a significant cause of mortality, including ~20% of all deaths in Western societies (3). It is estimated that 5 million cases of SCD occur worldwide each year. Also, 50% of all deaths associated with cardiovascular disease are caused by SCD (2). Although SCD rates range from 50–100 deaths per 100,000 people in the general population, there are differences in SCD rates comparing different age groups (2). According to a study conducted in Denmark, adults aged 35–49 years have 9.4 times the risk of dying from SCD compared to younger populations aged 1–35 years (4). Coronary artery disease (CAD) is responsible for 70-75% of all SCD cases in the population over the age of 35 (2, 5). In people aged <35 years, the rate of SCD is approximately 1–2 per 100,000 people in the general population (2, 6). The etiology of SCD varies and is highly dependent on the demographic characteristics of the patients included in the studies (7). A large number of SCDs at a younger age (<35 years old) have an unexplained etiology and potential underlying genetic basis (1, 8). Furthermore, the etiology of SCD in this group includes hypertrophic cardiomyopathy (HCM), dilated cardiomyopathy (DCM), arrhythmogenic right ventricular cardiomyopathy (ARVC), myocarditis, and different kinds of arrhythmogenic disorders including Brugada syndrome (BrS), congenital long-QT syndrome (LQTS), and catecholaminergic polymorphic ventricular tachycardia (CPVT) (6, 9).

SCD is a particularly traumatic event in seemingly healthy young athletes. Such deaths have a strong impact on the public because being an athlete is considered a healthy habit that prolongs and improves the quality of life.

CAD is the leading cause of SCD among athletes older than 35 years (10). Screening in that age group is much easier because CAD develops gradually throughout one's lifetime and is manifested by specific cardiac symptoms that alert the athlete. The situation is completely different among athletes under the age of 35 where most SCDs are caused by underlying heart disease triggered by intense exertion (8, 11).

Studies conducted in France (12) and the USA (11, 13) showed that young adults who are engaged in sports activities had 3.7–4.5 times the risk of SCD compared to age-matched young adults who were not athletes. Moreover, according to a study conducted in Italy (14), young adults who were not athletes had 0.41 times the rate of SCD compared to age-matched young adults who were athletes.

The cause of SCD is often associated with inherited or congenitally acquired mechanisms that provide the basis for the onset of malignant ventricular arrhythmia (15). HCM and ARVC are the most common causes of SCD in this age group (16, 17).

Other heart diseases that may also lead to SCD include myocarditis, dilated cardiomyopathy, congenital coronary anomalies, mitral valve prolapse, premature atherosclerotic coronary artery disease, WPW syndrome, etc. (17, 18). In a large number of SCD caused deaths, the autopsy findings show a normal cardiac structure, without any anomaly that would indicate pre-existing heart disease. In such cases, the cause of SCD is most commonly related to inherited cardiac ion-channel defects (channelopathies) which are responsible for the electrical activity of the heart (17, 19, 20).

### Research Aim

Our investigation sought to identify genetic variants in a family sample in which two individuals under 25 years-of-age died from SCD. The purpose of researching the genetic variants in this family lies in the fact that the brother (IV:4) and cousin (IV:2) of the proband (IV:3) play professional football, but is also important in first-degree relatives who are non-athletes and with potential evidence of significant genetic variants present in the family, prophylaxis could be performed in the form of more frequent thorough cardiac examinations and potential ICD implantation in high-risk individuals.

### Case Background

The proband (IV:3) was a professional footballer who suffered an SCD at the age of 23. Over a 2 year period, he lost consciousness 3 times playing in different professional clubs. After each syncope, all clinical examinations and complete cardiac diagnostics were performed. Due to the lack of a pathological finding, he was given a green light each time to return to the football fields. It is also important that his first cousin once removed (III:1) died at the age of 19 by collapsing abruptly on the football field during a match.

## Materials and Methods

### Participants and Case Preparation

This study includes the family of eight of the deceased professional footballers who died from SCD. To join the study, participants signed an informed consent form. Family members included in the study underwent genetic testing at Invitae Corp. clinical diagnostic laboratory. Knowing that the 2020 APHRS/HRS expert consensus statement indicates the required clinical examinations in the closest kin of the deceased, they were invited for a thorough cardiologic assessment. Due to the family's unwillingness to undergo further clinical examinations, only a part of the data was available. However, the proband's brother (IV:4), who is a professional football player, underwent a thorough clinical assessment including exercise test, holter ECG, echocardiography and ECG in order to determine possible underlying heart disease in line with the APHRS/HRS statement, which could potentially increase the risk of SCD (21). The results of his exercise test showed rare monomorphic ventricular extrasystoles during the first 3 min, later blocked with higher heartbeat frequency. Besides that, there was nothing pathological in the test. Holter ECG was recommended to all family members but was performed only in the same brother as mentioned above (IV:4). There were no pathological findings. Echocardiography didn't show any pathological findings. Echocardiography was also done in the father (III:4) and didn't show any pathological findings. An interpretation of the ECG findings of all family members is provided below.

Although the proband passed away 8 years ago, his family is still deeply saddened. Further investigations remind them of a tragic event therefore they are not prone to additional analysis.

### Cardiology Genetic Testing

Peripheral blood or saliva was processed and evaluated by Next Generation Sequencing (NGS). Each gene was targeted with oligonucleotide baits (Agilent Technologies, Santa Clara, CA; Roche, Pleasanton, CA; IDT, Coralville, IA) to capture all coding exons, plus 10–20 bases of flanking intronic sequences, and non-coding regions of clinical interest. Baits were balanced to obtain a minimum of 50× and an average of 350× depth-of-sequence read coverage. A bioinformatics pipeline was utilized that incorporated both standard and custom algorithms to identify single-nucleotide variants, small indels, large indels, structural variants with breakpoints in target sequences, and exon-level copy number variants (CNVs). In addition to standard GATK-based alignments and analysis, validated coverage-based CNV detection algorithms designed to flag possible split-read signals were applied. Once verified, the variant call format was updated and interpreted.

Variants were classified using a point-based scoring system congruent with the system for grading evidence for pathogenicity as recommended by the American College of Medical Genetics and Genomics (22). Orthogonal confirmation of CNVs was performed using gene-centric array-CGH. All testing for the 294-gene panel was performed at Invitae Corp., which is accredited by the Clinical Laboratory Improvement Amendments and College of American Pathologists. The Invitae cardiology genetic test provides a comprehensive analysis of 294 genes involved in the pathogenesis of inherited cardiovascular conditions: arrhythmia, cardiomyopathy, hereditary musculoskeletal diseases, anatomical anomalies of the cardiovascular system, connective tissue disorders with cardiac involvement, familial hypercholesterolemia, pulmonary hypertension, and congenital heart disease.

### Review of Family Pedigree

**Figure 1:**
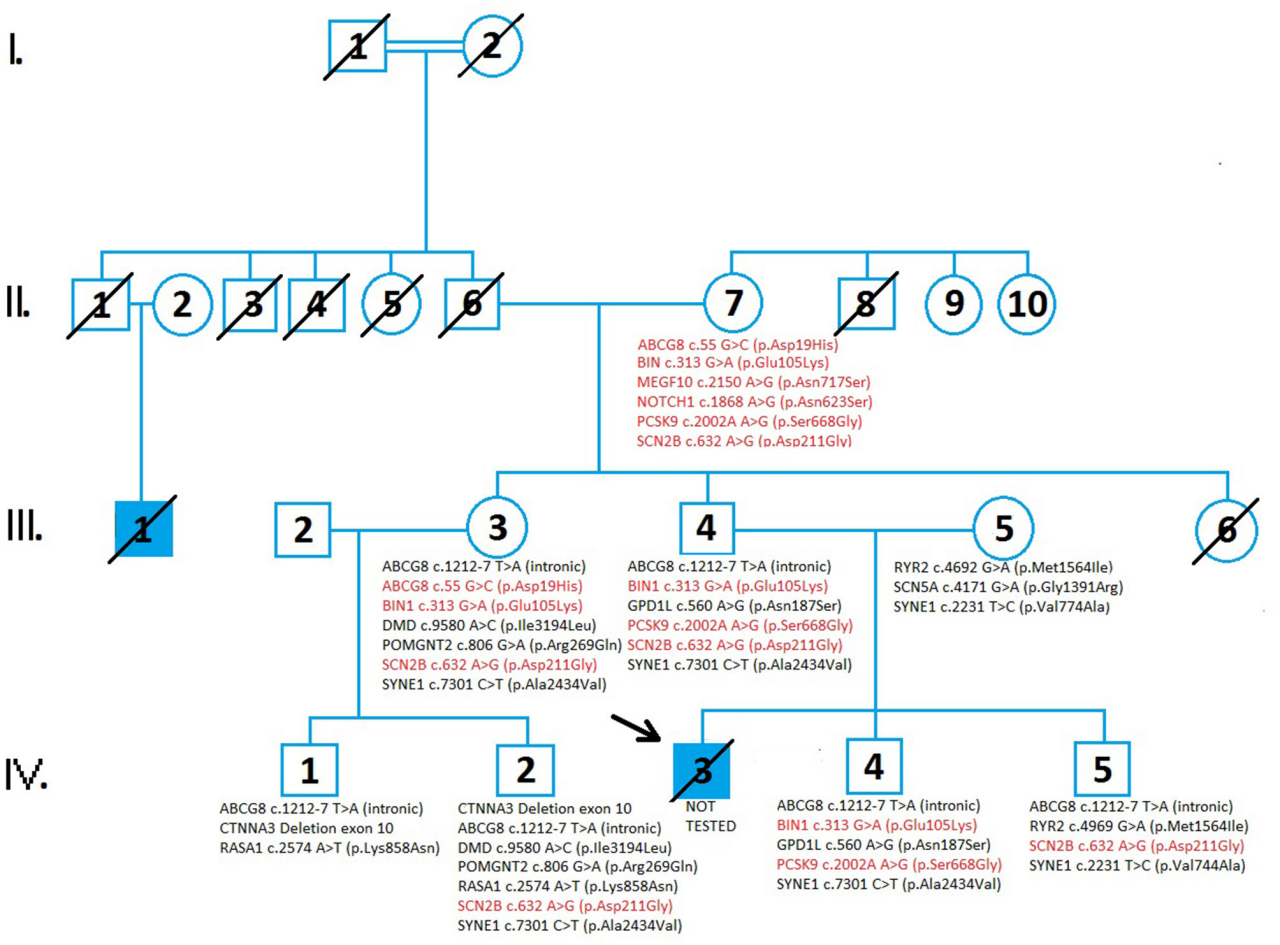
A pedigree analysis demonstrates the cases of sudden cardiac death and the results of genetic testing in four generations of family members. Circles indicate females while the squares indicate males. The diagonal line across the filled square indicated the patients died from sudden cardiac death. Genetic variants inherited from the proband's grandmother (II:7) are represented in red. Genetic variants inherited from the grandfather (II:6) of the proband are shown in black. Genetic testing in family members revealed variants of unknown significance (VUS) and there were no detected pathogenic variants in tested family members. The increased risk allele, ABCG c.55 G>C (p.Asp19His), is presented in II:7 and III:3. A comprehensive genetic test analyzed 294 genes involved in cardiac disease and conditions related to cardiac disease which can lead to sudden cardiac death.

The family pedigree consists of four generations (Figure 1). The first generation includes a great-grandmother (I:2) and great-father (I:1) of the proband (in which consanguinity can not be excluded) who had five children. All relatives of the family who could know the information about the degree of consanguinity have passed away and it is not possible to find out what degree of kinship it is. It is an isolated, closed population with only a few inhabitants. All inquiries to living relatives indicate the existence of consaguinity. All five children died in old age from an unknown cause. One of the great-uncles (II:1) had a son, (III:1) who died at the age of 19 by collapsing abruptly on the field during a football match. The grandfather (II:6) and grandmother (II:7) have two living children; aunt of the proband (III:3), father of the proband (III:4), while one of their daughters died at a young age from leukemia (III:6). Aunt (III:3) and her husband (III:2) have two living children (IV:1 and IV:2). Parents of the proband (III:4 and III:5) have two living sons, brothers of the proband (IV:4 and IV:5) and a proband (IV:3), who died at 23 years old from SCD.

### ECGs and Laboratory Blood Tests

A standard 12-channel ECG was performed on all participants. In order to better define possible additional cardiovascular risks (hypercholesterolemia) and based on results of genetic testing, biochemical blood tests were performed in the whole family. Biochemical blood tests included a complete blood count, lipid profile, and creatine kinase level.

## Results

### Analysis of Genetic Variants

The *ABCG8* gene (ATP binding cassette subfamily G member 8) encodes for sterolin-2, which in combination with sterolin-1 creates sterolin. Sterolin is a transporter protein responsible for eliminating plant sterols. *ABCG8* gene is related to autosomal recessive sitosterolemia, the disorder of cholesterol metabolism characterized by the accumulation of cholesterol in the skin, tendons, and various tissues. Polymorphisms in the *ABCG8* gene are associated with an increased risk of gallstones (23–25), and renal disease in patients with diabetes type 2 (26). Also, *ABCG8* gene polymorphism may contribute to the rapid onset of CAD in patients suffering from familial hypercholesterolemia (27, 28). A unique intronic variant in the *ABCG8* gene, c.1212-7 T>A (intronic) has been found in cousins (IV:2 and IV:1), the aunt (III:3), and brothers (IV:4 and IV:5) of the proband. According to the current prediction algorithm, variant c.1212-7 T>A (intronic) may disrupt the consensus splice site but it is not confirmed by transcriptional studies. An additional variant in the *ABCG8* gene; c.55 G>C (p.Asp19His), classified as an increased risk variant has been identified in the grandmother (II:7) of the proband. This variant has been associated with the development of gallstones.

The *BIN1* gene (Bridging integrator 1) belongs to the N-BAR domain, provides instructions for making membrane-associated protein, essential for biogenesis of plasma membrane invaginations (T-tubules) in muscle tissues, important for muscle contraction and relaxation. Initially, it was identified as a tumor suppressor protein (myc-interacting protein). The *BIN1* gene is associated with autosomal recessive and dominant centronuclear myopathy (CNM), characterized by muscle weakness (myopathy), abnormal localization of nuclei, and growth retardation. Several isoforms of the BIN1 protein are expressed with tissue and disease specificity and therefore some isoform BIN1 was discovered within cardiac transverse tubules (T-tubules) assumed to be important for cardiomyocyte homeostasis (calcium signaling) and is down-regulated in cardiomyopathy (29, 30). Heterozygous missense variant (VUS) c.313G>A (p.Glu105Lys) in the *BIN1* gene has been found in grandmother (II:7), father (III:4), aunt (III:3), and brother (IV:4) of the proband. The algorithm predicting the effect of missense changes on protein structure is contradictory at the moment, suggesting a “tolerated” or “probably damaging” impact.

The *CTNNA3* (catenin alpha 3) gene encodes a protein that belongs to the vinculin/alpha-catenin family, which has a role in cell-cell adhesion, specifically, binding plakophilins in cardiomyocytes. The *CTNNA3* gene is a new candidate gene for arrhythmogenic right ventricular cardiomyopathy (ARVC) (31). ARVC is a congenital heart disease that follows an autosomal dominant pattern, characterized by infiltration of adipose and fibrous tissue into the right ventricle and loss of myocardial cells, resulting in ventricular and supraventricular arrhythmias and RV dysfunction. A gross “in-frame” deletion variant (VUS) encompassing exone 10 of the *CTNNA3* gene results in a protein product lacking 31 amino acids. The functional significance of deleted amino acids is currently unknown and predicting algorithms are not available. *CTNNA3* gene variant was found in cousins (IV:1 and IV:2) of the proband.

The *DMD* (dystrophin) gene is the largest human gene producing cytoskeletal protein dystrophin, located primarily in muscles and in cardiac muscle, that enables the strength, stability, and functionality of myofibers (32). The *DMD* gene is associated with X-linked neuromuscular disorders Duchenne and Becker dystrophies, X linked dilated cardiomyopathy 3B, and familial cardiomyopathy. The underlying pathology of *DMD* related disease is the absence of essential muscle protein dystrophin caused by variants leading to an abnormal gene product. Dystrophin is partially functioning with a shorter protein product (33). A variety of variant types have been reported in the *DMD* gene. The identified heterozygous variant of uncertain significance (VUS) p.Ile3194Leu, is a rare point variant (missense change), with no clear impact on protein structure and/or function. In summary, all evidence is insufficient about the clinical significance of the variant and there is no indication that the variant causes disease. The variant has been identified in aunt (III:3) and her son (IV:2). Further analysis of family history data and detailed clinical examination did not reveal any signs of muscle weakness and muscular pathology in family members.

The *GPD1L* (glycerol-3-phosphate dehydrogenase 1 like) gene encodes a protein that catalyzes the conversion of sn-glycerol 3-phosphate to glycerone phosphate. The encoded protein binds the sodium channel, voltage-gated, type V, alpha subunit (*SCN5A*-coded Nav1.5) (34). It is expressed near the cell membrane and regulates the action potential of cardiomyocytes. Previous literature data suggest that genetic alterations in the *GPD1L* gene are related to cardiac rhythm disorders; Brugada syndrome, sudden infant death syndrome (SIDS), and long QT syndrome (35, 36). On the other hand, Hosseini et al. showed that only *SCN5A* gene alterations have definitive evidence in the pathogenesis of Brugada syndrome (37). The latest clinical study published in 2020 demonstrated that genetic alterations in the *GPD1L* gene led to decreased activation of the sodium channel and consequently early repolarization syndrome, one of the causes of sudden cardiac death (38). In this pedigree, father (III:4) and son (IV:4) share the same VUS in the *GPD1L* gene. According to the prediction algorithm, this variant is likely to be tolerated but additionally, functional and clinical studies for confirmation of its significance are needed.

The *MEGF10* (Multiple EGF-like domains 10) gene encodes multiple epidermal growth factor-like domain protein families, which contains 17 atypical EGF-like domains, each with eight cysteine residues (39). The encoded protein plays a role in cell adhesion, motility, and proliferation, and is a critical mediator of apoptotic cell phagocytosis as well as amyloid-beta peptide uptake in the brain. Expression of this gene may be associated with autosomal recessive early-onset minicore myopathy, areflexia, respiratory distress, and dysphagia (EMARDD) and is also associated with schizophrenia (40, 41). Specific missense change c.2150A>G (p.Asn717Ser) at codon 717 of MEGF10 protein has been identified as heterozygous in only one family member, grandmother (II:7) of the proband. The effect of sequence change on RNA splicing was predicted contradictory by PolyPhen to be “Benign” and by SIFT: to be “Deleterious.” The role of this variant in disease is uncertain since the available evidence is insufficient.

The *NOTCH1* (Notch 1) gene encodes for protein Notch1, a member of Notch family receptors. NOTCH is a key signaling pathway in the development of many tissues ensuring crosstalk between different types of cells, their physiological proliferation, differentiation, and cell self-destruction (apoptosis) (42). Despite its involvement in many key developmental systems, variants in the *NOTCH1* gene are mainly associated with autosomal dominant aortic valve disorder and Adams-Oliver Syndrome. Various cardiac disorders (BAV, aortic aneurysm, aortic coarctation), as well as the formation and progression of aortic valve calcification, were reported in correlation with *NOTCH1* variants (43). Heterozygous *NOTCH1* missense variant c.1868A>G (p.Asn623Ser) was also found in one family member, grandmother (II:7). Missense change on protein structure has a SIFT score deleterious and is rated as probably damaging by Poly-Phen 2. The contribution of this variant in predisposing disease remains uncertain and requires further evidence.

The *PCSK9* (proprotein convertase subtilisin/kexin type 9) gene is associated with familial hypercholesterolemia. Genetic variants of *PCSK9* are present in familial hypercholesterolemia and familial hypobetalipoproteinemia (44, 45).

A heterozygous missense variant in the *PCSK9* gene; c.2002A>G (p.Ser668Gly) has been found in the grandmother (II:7) and father (III:4) and brother (IV:4) of the proband. This variant does not disrupt protein function, but its significance is still not confirmed by functional studies.

The *POMGNT2* [Protein O-linked mannose N acetylglucosaminyltransferase 2 (beta 1,4)] gene encodes for endoplasmic reticulum (ER) – a resident protein that catalyzes the second step of the O-mannosyl glycosylation in the mucin-like domain of α-dystroglycan (46). Defect in glycosylation of α-dystroglycan led to a subgroup of muscular dystrophies, known as dystroglycanopathies. Genetic variants of *POMGNT2* are associated with limb-girdle muscular dystrophy (47). Heterozygous, *POMGNT2* missense variant c.806G>A (p.Arg.269Gln) was identified in two members, aunt (III:3) and cousin (IV:2). Missense change does not adversely affect protein structure and function, thus algorithm predictions by PolyPhen-2 “Benign” and SIFT “Tolerated” are aligned.

The *RASA1* (RAS p21 protein activator 1) gene encodes a protein called p120-RasGAP involved in the regulation of the RAS/MAPK signaling pathway from outside the cell to the cell's nucleus. Although its role is not entirely clear, it appears to be essential for the normal development of the vascular system. Variants in the *RASA1* gene are associated with autosomal dominant capillary malformation-arteriovenous malformations (CM-AVM) and Parker Weber syndrome (48, 49). Heterozygous, *RASA1* missense variant (VUS) c.2574A>T (p.Lys858Asn) has been identified in cousins (IV:2 and IV:1) of the proband. The specific missense change on protein structure and function has a SIFT score deleterious and is rated as probably damaging by Poly-Phen 2. The contribution of this variant in predisposing disease remains uncertain and requires further evidence.

The *RYR2* (ryanodine receptor 2) gene codes a protein called ryanodine receptor 2 involved in calcium transport within cells. *RYR2* gene alterations are involved in the pathogenesis of catecholaminergic polymorphic ventricular tachycardia, familial atrial fibrillation, and cardiomyopathy (20, 50). Mother (III:5) and son (IV:5) share the same VUS in the *RYR2* gene, c.4692G>A (p.Met1564Ile). In current literature, this variant has not been described and prediction tools gave inconclusive results regarding their clinical significance. This variant is transmitted from the maternal side so it could not be directly involved in both cases of SCD in this family but some additive effect of this variant is still unknown.

The *SCN5A* (sodium voltage-gated channel alpha subunit 5) gene codes the pore-forming alfa subunit of the primary cardiac channel. Genetic variants of *SCN5A* play role in Brugada syndrome, progressive familial heart block, Romano-Ward syndrome, sick sinus syndrome, familial atrial fibrillation, and familial dilated cardiomyopathy (51–53). In our study, a mother (III:5) without any cardiac condition had a heterozygous (VUS) variant, c.4171G>A (p.Gly1391Arg) in the *SCN5A* gene and it wasn't transmitted to a currently living offspring.

The *SCN2B* (Sodium Voltage-Gated Channel Beta Subunit 2) gene codes the beta II subunit of type II voltage-gated sodium channel. *SCN2B* gene alterations lead to autosomal dominant Brugada syndrome, atrial fibrillation, and sudden infant death syndrome (54). Heterozygous VUS has been found in all generations, including II:7, III:3, III:4, IV:2, and IV:5, suggesting an autosomal dominant mode of inheritance. This *SCN2B* variant, c.632A>G (p.Asp211Gly) in exon 4 has been reported to affect SCN2B protein function. The algorithm predicting the effect of sequence changes on RNA splicing suggests that this variant may create or strengthen a splice site. Unfortunately, this prediction is not yet confirmed by transcriptional studies.

The *SYNE1* (spectrin repeat-containing nuclear envelope protein 1) gene codes Syne-1 protein is mostly expressed in the cerebellum and involved in the coordination of movements. Their genetic alterations can lead to autosomal recessive cerebellar ataxia type 1, Emery-Dreifuss muscular dystrophy, and recently there are some reports of dilated cardiomyopathy in some patients (55, 56). Our study revealed two different VUSs. The heterozygous *SYNE1*: c.7301C>T (p.Ala2434Val) variant has been found in III:3, III:4, IV:2, and IV:4, while the heterozygous c.2231T>C (p.Val744Ala) variant was found in III:5 and IV:5. Both variants require further transcriptional studies to confirm their effect on protein function. There were no ataxia cases in this family. A summary of the genetic variants is provided in the table below (Table 1).

**Table 1:** List of genetic biomarkers recorded by INVITAE cardiology genetic test (SCD – sudden cardiac death, SNP – single nucleotide polymorphism, VUS – a variant of uncertain significance).

**Gene**	**Gene function**	**Association with SCD**	**ZYGOSITY**	**SNP/variant**	**Pathogenic variant or VUS**	**A family member with genetic variant**	**gnomADv2.1.1 frequency**	**V3.1 frequency**
ABCG8	Encodes a protein sterolin-2 which transports plant sterols	1. Sitosterolemia 2. Renal disease in patients with diabetes type 2 3. Enhancement of the effect of familial hypercholesterolemia 4. Gallstones	Heterozygous	c.1212-7 T>A (intronic)	VUS	III:3, III:4, IV:1, IV:2, IV:4. IV:5.	0.0001273	0.0001052
ABCG8	Encodes a protein sterolin-2 which transports plant sterols	1. Sitosterolemia 2. Gallstones 3. Renal disease in patients with diabetes type 2 4. Enhancement of the effect of familial hypercholesterolemia	Heterozygous	c.55 G>C (p.Asp19His)	Pathogenic variant	II:7, III:3	0.06640	missing VEP annotations
BIN1	Encodes a BIN1 protein that has a role in endocytosis and apoptosis	1. Skeletal muscle myopathy 2. Regulation of calcium homeostasis 3. Cardiomyopathy	Heterozygous	c.313 G>A (p.Glu105Lys)	VUS	II:7, III:3, III:4, IV:4	absent	absent
CTNNA3	Encodes a protein that belongs to the vinculin/alpha-catenin family	Arrhythmogenic right ventricular dysplasia	Heterozygous	Deletion exon 10	VUS	IV:1, IV:2	N/A	N/A
DMD	Encodes protein dystrophin -has a role in strengthening muscle fibers	1. X-linked dilated cardiomyopathy 2. Familial dilated cardiomyopathy 3. Duchenne muscular dystrophy	Hemizygous	c.9580 A>C(p.Ile3194Leu)	VUS	III:3, IV:2	0.00002480	0.00007145
GPD1L	Encoded a protein that catalyzes the conversion of sn-glycerol 3-phosphate to glycerone phosphate	1.Brugada syndrome 2. Brugada syndrome 2 3. Long QT syndrome 4. Sudden infant death syndrome	Heterozygous	c.560 A>G (p.Asn187Ser)	VUS	III:4, IV:4	0.000003977	absent
MEGF10	Encodes a member of the multiple epidermal growth factor-like domains protein family	1. Respiratory distress 2. Myopathy	Heterozygous	c.2150 A>G (p.Asn717Ser)	VUS	II:7	0.000178	0.0001248
NOTCH1	Encodes a protein called Notch1, a member of the Notch family of receptors	1. Critical congenital heart disease 2. Bicuspid aortic valve 3. Aortic aneurysm 4. Aortic coarctation 5. Formation and progression of aortic valve calcification	Heterozygous	c.1868 A>G (p.Asn623Ser)	VUS	II:7	0.00003260	absent
PCSK9	Encodes a protein that helps regulate cholesterol level in the blood	1. Familial hypercholesterolemia 2. Familial hypobetalipoproteinemia	Heterozygous	c.2002 A>G (p.Ser668Gly)	VUS	II:7, III:4, IV:4	0.00004790	0.00003941
POMGNT2	Encodes a protein with glycosyltransferase activity	Limb-girdle muscular dystrophy	Heterozygous	c.806 G>A (p.Arg269Gln)	VUS	III:3, IV:2	0.00002387	absent
RASA1	Encodes a protein called p120-RasGAP included in RAS/MAPK signaling pathway	1. Parkes Weber syndrome 2. Capillary malformation-arteriovenous malformation syndrome (CM-AVM)	Heterozygous	c.2574 A>T (p.Lys858Asn)	VUS	IV:1, IV:2	0.000007988	absent
RYR2	Encodes a protein called ryanodine receptor 2 involved in the regulation of calcium channels	1. Catecholaminergic polymorphic ventricular tachycardia (CPVT) 2. Arrhythmogenic right ventricular cardiomyopathy 3. Familial atrial fibrillation	Heterozygous	c.4692 G>A (p.Met1564Ile)	VUS	III:5, IV:5	0.00008902	0.00003287
SCN5A	Produces a protein essential for the regulation of sodium channels	1. Romano-Ward syndrome 2. Brugada syndrome 3. Progressive familial heart block 4. Sick sinus syndrome 5. Familial atrial fibrillation 6. Familial dilated cardiomyopathy	Heterozygous	c.4171 G>A (p.Gly1391Arg)	VUS	III:5	0.00003603	absent
SCN2B	Encodes beta 2 subunits of type II voltage-gated sodium channel	1. Brugada syndrome 2. Atrial fibrillation	Heterozygous	c.632 A>G (p.Asp211Gly)	VUS	II:7, III:3, III:4, IV:2, IV:5	0.000007954	0.00001972
SYNE1	Encodes an SYNE-1 protein present in Purkinje cells responsible for coordinating movement	1. Emery-Dreifuss muscular dystrophy 2. Dilated cardiomyopathy	Heterozygous	c.7301 C>T (p.Ala2434Val)	VUS	III:3, III:4, IV:2, IV:4	absent	missing VEP annotations
SYNE1	Encodes an SYNE-1 protein present in Purkinje cells responsible for coordinating movement	1. Emery-Dreifuss muscular dystrophy 2. Dilated cardiomyopathy	Heterozygous	c.2231 T>C (p.Val744Ala)	VUS	III:5, IV:5	absent	absent

### Pedigree Analysis

The pedigree analysis encompassed available data from four generations of the family (Figure 1). Available data included data on health conditions from medical history, causes of death, results of laboratory testing, and genetic testing results.

Grandmother (II:7) has hypertension and increased levels of blood cholesterol. Her genetic test revealed six variants of unknown significance that are related to cholesterol metabolism (*ABCG8, PCSK9*), muscle contraction (*BIN1*), tissue development, and apoptosis (*NOTCH1, MEGF10*) as well as sodium channel in cardiac muscle (*SCN2B*).

In the third generation, genetic testing was performed on the mother (III:5), father (III:4), and aunt (III:3). Father (III:4) and aunt (III:3) had the same variants in genes: *ABCG8, BIN1, SCN2B*, and *SYNE1* (related to Emery-Dreifuss muscular dystrophy with cardiomyopathy, cardiac conduction defects, and cerebellar ataxia). Person III:3 is a compound heterozygote for ABCG8 gene variants with observed elevated blood cholesterol, potentially induced by the variants genotype. However, her BMI is 48.4 therefore the increase in cholesterol levels can be attributed to her diet and lifestyle habits. Additional variants in aunt were present in *DMD* (Duchenne muscular dystrophy), *POMGNT2* (dystrophy-dystroglycanopathy type A8) that are not involved in cardiac pathology. An additional variant in father (III:4) was present in the *GPD1L* gene, involved in the pathogenesis of early repolarization syndrome, Brugada syndrome type 2 and sudden cardiac death in children. Genetic testing in the mother (III:5) revealed variants in the *RYR2* gene related to cardiomyopathy, autosomal catecholaminergic polymorphic ventricular tachycardia, arrhythmogenic dysplasia of the right ventricle. Additional variants were present in *SYNE1* and *SCN5A* genes related to various cardiac conduction defects (Brugada syndrome, long QT interval type 3, and cardiomyopathy).

In the fourth generation, genetic testing was performed in brothers (IV:4 and IV:5) of the proband. They share the same variants in *ABCG8* and *SYNE1* gene. Additional variants were present in *BIN1, GPD1L, PCSK9*, and *RYR2*, and *SCN2B*.

According to the test results, cousins (IV:1 and IV:2) have the same variants in *ABCG8* (intronic), *CTNNA3*, and *RASA1* genes, involved in the pathogenesis of arrhythmogenic right ventricular cardiomyopathy (*CTNNA3*) and *RASA1* (capillary malformations, Park Weber syndrome). Also, additional variants of *DMD, POMGNT2, SCN2*, and *SYNE1* gene were found in IV:2.

### Interpretation of Participants' Laboratory Blood Tests and ECGs

The ECGs of all study participants showed an orderly finding of heart action, without any pathological changes suggesting heart disease (Table 2). Complete blood counts in all participants showed no major deviations from normal values. Blood cholesterol was increased in grandmother (II:7), mother (III:5), father (III:4), and cousin (IV:1). Interestingly, the highest blood cholesterol level has been found in the mother of an index patient who doesn't have any genetic alterations in these cholesterol metabolism-related genes. Also, it is important to consider a high body mass index in these patients, which contributes to alterations in cholesterol metabolism and blood levels. III:5, II:7, IV:1, and III:3 have an increased cholesterol ratio that matches their phenotype. III:5 and IV:1 have an increased creatine kinase which can also be associated with being overweight (Table 3).

**Table 2:** Description of ECG records of all participants.

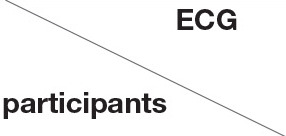	**Rhytm**	**Electrical axis**	**ST-T interval description**	**QT/QTc**	**Additional findings**
					
IV:4	Sinus rhythm 50/min	Intermediate	No changes	440/402 msec	Incomplete RBBB
IV:5	Sinus rhythm 61/min	Intermediate	No changes	442/427 msec	
IV:2	Sinus rhythm 45/min	Intermediate	No changes	476/411 msec	PVC
IV:1	Sinus rhythm 99/min	Intermediate	No changes	363/419 msec	
III:5	Sinus rhythm 67/min	Intermediate	No changes	404/427 msec	
III:4	Sinus rhythm 48/min	Intermediate	No changes	500/447 msec	
III:3	Sinus rhythm 66/min	Intermediate	No changes	393/406 msec	
II:7	Atrial fibrillation with ventricular response of 91/min	Intermediate	No changes	338/386 msec	

*(RBBB- right bundle branch block, PVC- premature ventricular contraction)*.

**Table 3:** Findings in laboratory analysis of blood and association with BMI.

**Laboratory findings and BMI**	**Total cholesterol**	**LDL**	**VLDL**	**HDL**	**Triglycerides**	**Cholesterol ratio**	**CK**	**BMI**
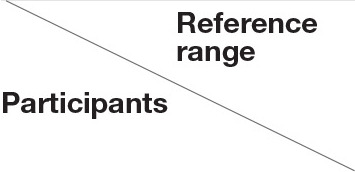	<5,0 mmol/L	<3,0 mmol/L	0,1-0,5 mmol/L	W >1.2mmol/LM = 1,0-1,9 mmol/L	<1,7 mmol/L	<3,5	W <153 U/L M <177 U/L	<25 kg/m^2^
								
III:5	**7.8**	/	/	**1.2**	**8.7**	**6.5**	**161**	**31,6**
II:7	**6.5**	**4.0**	**0.8**	1.7	**1.7**	**3.8**	51	**32,2**
IV:1	**7.1**	**5.0**	**0.8**	1.3	**1.8**	**5.5**	**284**	**46,8**
III:3	**5.9**	**3.6**	**1.0**	1.3	**2.1**	**4.5**	102	**48,4**
IV:5	3.9	1.9	**0.8**	1.2	**1.8**	3.3	128	**31,1**
IV:4	4.5	2.5	0.4	1.5	1.0	3.0	73	**25,6**
IV:2	4.3	1.9	0.4	**2.1**	0.8	2.0	**240**	23,8
III:4	**5.7**	**3.5**	/	**2.0**	1.4	2.9	107	**42,0**

*Elevated values are bolded*.

*Fields with “/” represent uncalculated values due to technical difficulties. (LDL-low density lipoprotein, VLDL-very low-density lipoprotein, HDL-high density lipoprotein, CK- creatine kinase, BMI-body mass index)*.

## Discussion

### Interpretation of Genetic Variants

SCD is a catastrophic event not only for the patient and his family but also for the medical staff and the general public. According to the CDC (Center for Disease Control), approximately, more than 2,000 people in the USA experience sudden cardiac death each year. Following recommendations of the European Society of Human genetics (57) our study has been conducted by a multidisciplinary team and took all relevant family history data, clinical investigation and cardiac genetic screening test. In clinical practice, a family history of SCD requires careful medical attention and evaluation. In the era of genomic medicine genetic testing and detection of pathogenic variants enables accurate diagnosis and exact risk assessment for the whole family (57, 58). Unfortunately, in this manuscript as well as in many SCD cases biological samples from deceased patients for genetic analysis were not taken and it was is not possible to define a diagnosis, calculate risk for family members, and provide appropriate medical care.

In this manuscript, we describe four generations of the family with two cases of SCD at an early age.

Pedigree analysis in this family revealed alterations in seven genes related to various cardiac conditions, mostly involved in cardiac rhythm disorders (*GPD1L, RYR2, SCN5A, SCN2B*), structural abnormalities that could involve cardiac muscle (*CTNNA3, DMD*), and regulation of calcium homeostasis in cardiac muscle *(BIN1)* but according to our updated knowledge, none of this variants were pathogenic. In order to get a more comprehensive insight into SCD and potential pathophysiological mechanisms, our analysis included additional genes related to glycosylation (α-dystroglycan, *POMGNT2*) and the development of vascular abnormalities (*RASA1*). In latter cases, the analysis did not reveal pathogenic variants. Considering that SCD at an early age is usually the result of an unrecognized cardiac conduction defect, we paid special attention to the analysis of genes involved in cardiac rhythm disorders. According to modern cardiology concepts (59), the pathogenesis of cardiac arrhythmia includes abnormalities in ion channel activity, cellular signaling, and structural abnormalities mediated by a large number of genes.

As can be seen from the pedigree analysis same variants; *SCN2B* c.632A>G (p.Asp211Gly) and *BIN1* c.313G>A (p.Glu105Lys) were present in II generation (II:7), III generation (III:3 and III:4), and their offspring in IV generation (IV:4 and IV:2). In these patients, there were no cardiac events and the results of an ECG and heart ultrasound were normal.

Analysis of other variants in this pedigree showed a variant of unknown significance; *GPD1L* c.560A>G (p.Asn187Ser) present in the father (III:4) and brother (IV:4) of the proband. This variant wasn't present in the II generation (II:7) and other family members suggesting that this variant originates from the paternal side of the II generation [grandfather (II:6) and his brother (II:1)]. Father's (III:4) cousin (III:1) also experienced SCD at an early age and there is a 50% possibility that he had this variant also. Unfortunately, we did not have a possibility to perform genetic testing in person III:1 so we can not confirm this hypothesis. Also, it is very important to emphasize that the proband might have inherited *SCN5A* c.4171 G>A (p.Gly1391Arg) genetic variant from his mother (III:5). She represents the only carrier of this genetic variant in the whole pedigree. The fact that proteins encoded by *SCN5A* and *GPD1L* are included in the same regulatory pathway, in which *SCN5A* encodes sodium channel, voltage-gated, type V, alpha subunit, and *GPD1L* encodes an intracytoplasmic membrane-bound enzyme bonded to that sodium channel, may indicate that there was a chance for the additive effect of the two genetic variants, ie digenic inheritance (34–36). It has been proven that 30% of the causes of Brugada syndrome originate in the polymorphism of the *SCN5A* gene. Genetic variants of other genes, including *GPD1L, SCN1B, SCN2B, SCN3B, RANGRF, SLMAP, KCNE3, KCNJ8, HCN4, KCNE5, KCND3, CACNA1C, CACNB2B, CACNA2D1*, and *TRPM4*, may also underlie Brugada syndrome. These genes encode proteins responsible for cardiac sodium, potassium, and calcium channels, as well as proteins involved in the circulation or regulation of these channels (52). When the enzymatic activity of the protein encoded by *GPD1L* is decreased, levels of glycerol 3-phosphate are higher. It activates the *GPD1L*-dependent *SCN5A* phosphorylation pathway. Consequently, sodium current may be decreased. Also, an imbalance of NAD(H) induced by *GPD1L* can result in a reduced-sodium current. Combined with a possible co-present defective protein encoded by *SCN5A*, it may have increased the chance of developing Brugada syndrome (60). Given that the proband had a 2-year history of cardiac symptoms, where he had syncope on three occasions and after each complete cardiac treatment had a proper finding and received the green light to continue playing professional football, it can be assumed that he did not suffer from any structural heart disease which would be seen during the routine ultrasound examination.

Also, according to Snir et al. a regular ECG finding does not rule out the existence of Brugada syndrome, because many patients have an intermittent Brugada syndrome ECG pattern (61).

Various triggers of Brugada syndrome are mentioned in the literature including fever, lithium, antidepressants, sodium channel blockers, and exercise (62–64). Therefore, diagnosis is difficult, and it is easy to overlook the existence of a Brugada pattern. This may explain the fact why the proband did not have any pathological findings on cardiac examinations.

Although the genetic variants *PCSK9* c.2002A A>G (p.Ser668Gly), *ABCG8* c.1212-7 T>A (intronic) and *ABCG8* c.55 G>C (p.Asp19His) present in the grandmother (II:7) and the father (III:4) of the proband are responsible for hereditary hypercholesterolemia, the proband died at the age of 23 without any visible signs of hypercholesterolemia including xanthoma, xanthelasma, jaundice. Therefore, the etiology of CAD in the event of SCD can be ruled out.

The presence of consanguinity in grandfather's side of first-generation increases the risk for the presence of various inherited disorders including cardiac conduction abnormalities. Taken together, the presence of consanguinity in this part of the family, as well as the presence of a unique variant in the *GPD1L* gene not present in the grandmother (II:7) indicates that SCD could be related to genetic alterations in the grandfather (II:1) and his siblings /relatives. Unfortunately, grandfather (II:1) and his relatives died so this assumption cannot be confirmed by genetic testing. Additional functional studies are needed to clarify the role of *GPD1L* c.560A>G (p.Asn187Ser) variant in the pathogenesis of Brugada syndrome.

In the third-generation, the mother (III:5) had VUS variants in two genes involved in cardiac rhythm disorders; *SCN5A* and *RYR2*, and she didn't have any cardiac pathology condition. *SCN5A* genetic alterations are present in 30% of all Brugada syndrome cases while the *RYR2* genetic alterations are reported because of catecholaminergic polymorphic ventricular tachycardia, atrial fibrillation, and arrhythmogenic right ventricular cardiomyopathy.

Current literature data emphasize the high variability of disease phenotype in patients with the same pathogenic variant (*SCN5A*), even within the same family. There are patients with malignant arrhythmias, SCD and on the other hand, there are patients without any clinical signs. Reduced or incomplete penetrance in these families has been observed (65). Also, disease expressivity and severity are regulated by individual-specific factors related to other cardiac ion channels that regulate cardiac action potential. Recent studies suggest that single nucleotide polymorphisms, copy number variations, and their combination can modulate disease expressivity and severity in patients with *SCN5A* variants.

Additional non-genetic modifiers such as gender, age, tobacco, drug and alcohol use, medication, exercise, fever, comorbidities, and lifestyle are also important determinants of disease severity. It is well known that individual *SCNA5* variant carriers develop long QT intervals early in life (at birth) while Brugada syndrome is seen later in life (66). Alcohol, fever, tobacco, exercise, drug, and some medication may modulate the electrical activity of the cardiac channel directly or indirectly and trigger arrhythmia and cardiac events. Obesity and hypertension are related to metabolic disturbances (dyslipidemia) and progressive myocardial remodeling that result in electrical, homeostatic, and structural alterations. All these changes act synergistically and can precipitate cardiac events.

Interestingly, both parents of the proband had variants in two different genes involved in cardiac rhythm diseases. It is assumed that some additive effects of these variants in cardiac disease pathology cannot be excluded. Ultimately, the interpretation of variants depends on their frequency in the population and ethnic background that also has to be taken into consideration.

Lack of visible ECG abnormalities and absence of structural cardiac disorder on a routine ultrasound could obscure cardiac structural or conduction defects in many patients with a previous history of cardiac syncope and family history of sudden cardiac death. Genetic testing in these patients enables the detection of various genetic variants and could modify routine diagnostic workup. Detection of pathogenic variants in genes involved in cardiac conduction (*GPD1L, RYR2, SCN5A, SCN2B*) and structural (*CTNNA3*) defects indicates a more comprehensive approach in the diagnostic workup and requires additional testing; ajmaline provocation tests (Brugada syndrome), electrophysiology cardiac studies as well as magnetic resonance and echocardiographic strain imaging.

Even though our case did not involve mitochondrial DNA analysis, heart diseases that can lead to SCD can be inherited by mitochondrial DNA and this type of inheritance should be considered in further research (67).

As well as in many other cases of SCD, the absence of an autopsy in deceased individuals and lack of available biological samples for genetic testing is the main limitations of our study. Despite the initiative of European Council and Recommendations (68) that enable clear criteria when autopsy is performed, there are still large variations among countries. The lack of available biological samples for genetic testing data does not allow any insight into the genetic basis of SCD in deceased individuals. Recent studies showed a diagnostic yield of 40% in cases where a multidisciplinary team approach conducted postmortem genetic testing, family and clinical investigation (69, 70). In the circumstances of our legislation, our study tried to elucidate the genetic basis of SCD in this family using all available resources. Although we did not detect pathogenic variants in this family that could explain SCD cases, we found many variants whose effect is still unknown and requires additional investigations.

### Future Perspective

Based on a comparison between genes of living relatives with the genes of the deceased, significant gene variants could be identified and associated with an increased risk of SCD. Although current clinical guidelines do not recommend genetic testing in patients without symptoms (syncope, arrhythmia) it is not possible to ignore opportunities and benefits (non-invasive method compared to various provocation tests, detection of pathogenic variant) of genetic testing. Since genetic testing is a non-invasive method, it should be a routine part of the diagnostic workup in patients with cardiac syncope/family history of sudden cardiac death. Based on genetic testing results it is possible to change diagnostic workup using electrophysiological studies and more detailed cardiac imaging studies that enable early detection of cardiac pathology. Such a discovery would provide a revolutionary new indication at the level of genetics, that would provide the possibility of ICD prophylaxis in those patients who haven't yet developed heart disease. Based on risk factors, genotype, and clinical examination results, patients could be stratified into specific disease phenotype groups which would facilitate further patient management. The purpose of this kind of approach is in reducing the incidence and consequently the mortality from SCD. Genetic testing in these patients enables detection of inherited pathogenic variants, but there is also a high probability of uncertain findings, that cannot explain SCD in these families. Appropriate genetic counseling, before and after testing, provides at-risk relatives with relevant information on genetic basis of SCD, possibilities of genetic testing and its impact on further management. During pre-testing genetic counseling, all possible outcomes of genetic testing should be discussed and explained in detail to the patient. In this way, the level of psychological stress is reduced to a minimum.

Genetic testing inherently increases costs of management of relatives who are at risk of SCD, however this cost is lower than medical-cardiovascular regular check-ups every 3–5 years (71). Considering all aspects of SCD management, benefits and limits of genetic testing and limited knowledge on SCD, our study encourages genetic testing in at-risk relatives as a powerful tool that could elucidate cause of SCD and give valuable data needed for appropriate clinical management. This approach has been successfully implemented in diseases where genetic background has significant pathogenetic role. Breast cancer diagnostics and management showed suitable example of this approach. In these patients, use of genetic testing in combination with relevant clinical, radiological and laboratory data has significantly influenced diagnostic and therapeutic procedures (72).

## Conclusion

In conclusion, our pedigree analysis did not find any pathogenic or likely pathogenic variant according to existing data that could give a valuable explanation of SCD in this family. Both parents had variants in several cardiac-related genes, but their clinical significance is still uncertain and we cannot ignore possible causal relation in the pathogenesis of SCD. For this reason, additional functional *in vitro*/*in vivo* studies are necessary to determine the importance of these variants and related risks. This family study emphasizes the importance of a systematic approach in the diagnostic workup of these patients and their relatives. A detailed approach requires concise family history data, results of clinical testing and laboratory data, and of taking blood samples for genetic testing in every case of SCD. Furthermore, large family cohorts, segregation analysis, additional functional studies of variants, detailed family history, and clinical examination are important for a better definition of disease-related genes and translation variants in clinically relevant data. We should also be aware that the variants of uncertain significance are prone to reclassification with further analysis and should be careful when interpreting their clinical significance.

## Data Availability Statement

The raw data supporting the conclusions of this article will be made available by the authors, without undue reservation.

## Ethics Statement

The studies involving human participants were reviewed and approved by Ethics Committee of the St. Catherine Specialty Hospital. The patients/participants provided their written informed consent to participate in this study.

## Author Contributions

DP: conceptualization. DP, LO, VP, JĆ, and AS: data curation. DP, LO, VP, JĆ, JŠ, VR, ŠM, RN, MV, SA, TS, VMa, VMo, AS, JM, and JB: roles/writing—original draft. DP, VP, VMa, VMo, and JB: writing—review & editing. All authors contributed to the article and approved the submitted version.

## Conflict of Interest

The authors declare that the research was conducted in the absence of any commercial or financial relationships that could be construed as a potential conflict of interest.
